# High Power Energy Storage via Electrochemically Expanded and Hydrated Manganese-Rich Oxides

**DOI:** 10.3389/fchem.2020.00715

**Published:** 2020-08-18

**Authors:** Shelby Boyd, Natalie R. Geise, Michael F. Toney, Veronica Augustyn

**Affiliations:** ^1^Department of Materials Science and Engineering, North Carolina State University, Raleigh, NC, United States; ^2^Department of Chemistry, Stanford University, Stanford, CA, United States; ^3^Stanford Synchrotron Radiation Lightsource, SLAC National Accelerator Laboratory, Menlo Park, CA, United States

**Keywords:** aqueous electrolyte, manganese oxide, interlayer, confined water, electrochemical capacitor

## Abstract

Understanding the materials design features that lead to high power electrochemical energy storage is important for applications from electric vehicles to smart grids. Electrochemical capacitors offer a highly attractive solution for these applications, with energy and power densities between those of batteries and dielectric capacitors. To date, the most common approach to increase the capacitance of electrochemical capacitor materials is to increase their surface area by nanostructuring. However, nanostructured materials have several drawbacks including lower volumetric capacitance. In this work, we present a scalable “top-down” strategy for the synthesis of EC electrode materials by electrochemically expanding micron-scale high temperature-derived layered sodium manganese-rich oxides. We hypothesize that the electrochemical expansion induces two changes to the oxide that result in a promising electrochemical capacitor material: (1) interlayer hydration, which improves the interlayer diffusion kinetics and buffers intercalation-induced structural changes, and (2) particle expansion, which significantly improves electrode integrity and volumetric capacitance. When compared with a commercially available activated carbon for electrochemical capacitors, the expanded materials have higher volumetric capacitance at charge/discharge timescales of up to 40 s. This shows that expanded and hydrated manganese-rich oxide powders are viable candidates for electrochemical capacitor electrodes.

## Introduction

Electrochemical energy storage plays ever-increasing roles in our daily lives by powering everything from portable electronics to electric vehicles. Electrochemical capacitors (ECs) are a class of energy storage devices with intermediate energy and power between those of batteries and dielectric capacitors. Commercialized ECs are highly attractive for diverse applications requiring reliability, large currents, and high energy efficiency. They exhibit specific energy of up to 10 Wh/kg, specific power of up to 30 kW/kg, typical charge/discharge timescales on the order of seconds, and lifetimes of up to ~1,000,000 cycles (Conway, 1999; Nybeck et al., 2019). Most commercialized ECs store energy by the rapid (within seconds) formation of the electric double layer at the electrode/electrolyte interface. This mechanism can give rise to specific capacitance values of up to 150 F/g when the electrolyte ion size matches the average pore size of the electrode material (Largeot et al., 2008). Another capacitive energy storage mechanism, termed pseudocapacitance, uses fast and reversible redox reactions in materials such as transition metal oxides and can lead to capacitances >150 F/g (Fleischmann et al., 2020).

There is a need to increase the energy density and reduce the cost of ECs to enable their use for broader applications. However, this requires the development of new electrode materials that exhibit high energy storage performance and are made from abundant elements via scalable synthesis processes. Thus far, the search for new EC materials has primarily focused on the synthesis of nanostructured materials. This improves the power density by increasing the interfacial area between the electrode and electrolyte, and reducing the solid-state diffusion distance within the particles (Okubo et al., 2007; Rauda et al., 2014; DeBlock et al., 2019; Li et al., 2020). However, the high surface area of nanostructured particles can lead to undesirably low volumetric capacitance electrodes and increased parasitic side reactions with the electrolyte (Palacín et al., 2016; Augustyn and Gogotsi, 2017). Additionally, the decrease of tap density with particle size lowers the volumetric capacity of electrodes made with small particles (Jouanneau et al., 2003; Li et al., 2019). Therefore, there is a need for high-power energy storage materials that match the rate performance of high surface area materials but exhibit higher volumetric capacitance, as well as scalable strategies to synthesize such materials.

Commonly studied materials for ECs include transition metal oxides with hydrated interlayers. Oxides such as V_2_O_5_·*x*H_2_O, WO_3_·2H_2_O, and birnessite (typical formula K_*x*_MnO_2_·*n*H_2_O) show pseudocapacitive behavior in aqueous electrolytes due to reversible cation intercalation (Nam et al., 2015; Sai Gautam et al., 2016; Mitchell et al., 2017; Xiong et al., 2017; Wang et al., 2018; Wu et al., 2019). The interlayer water in these materials is hypothesized to enable fast energy storage by buffering structural changes during cycling, reducing transition metal dissolution into the electrolyte, and/or improving the interlayer diffusion kinetics of intercalating cations (Zhu et al., 2017; Wang et al., 2018; Liu Z. et al., 2019). In particular, birnessite manganese oxides are promising EC materials due to the abundance and safety of manganese. However, their low crystallinity and frequently nanoscale morphology result in low material utilization and packing density, and therefore low volumetric capacitance in thick electrodes (Wu et al., 2015; Wang et al., 2017; Liu et al., 2018). An attractive alternative is developing material synthesis techniques to produce micron-scale transition metal oxide particles with hydrated interlayers, which could lead to high volumetric capacitance EC electrodes made via traditional slurry electrode manufacturing techniques.

To address this, we propose the use of electrochemically expanded and hydrated manganese oxides based on P2 oxides of the formula Na_0.67_*M*O_2_. Here, *M* is a combination of manganese and other transition metals and P2 indicates the prismatic (P) coordination of Na^+^ and …ABBA… stacking of the oxygen layers. The high temperature (~900°C) synthesis of the P2 oxides leads to micron-scale primary particles with surface areas of ~1 m^2^/g (Yabuuchi et al., 2014). P2 Na_0.67_*M*O_2_ are widely studied for non-aqueous sodium ion battery cathodes, but often exhibit poor cyclability due to the Jahn-Teller distortion and dissolution of the Mn atoms during electrochemical cycling, and poor electronic and ionic conductivity (Clément et al., 2015; Clement et al., 2016; Nam et al., 2015; Liu C. et al., 2019). However, these materials could be promising as EC electrode materials due to their tendency to form hydrated phases under ambient conditions and upon electrochemical cycling in aqueous electrolytes (Lu and Dahn, 2001; Buchholz et al., 2014; Boyd et al., 2018). In our previous work, we established that this water intercalation causes a 25% *c*-axis expansion and a phase transformation to a birnessite-like structure, which subsequently exhibits capacitive electrochemical behavior (Boyd et al., 2018). While these electrodes experience a severe capacity decline from active material detachment due to the large structural change, the capacitive response of the remaining expanded, hydrated oxide led us to consider whether it was possible to perform the expansion prior to electrode assembly. The resulting expanded micron-sized particles of manganese-rich oxide with hydrated interlayers could then be used for high rate capability and high volumetric energy density EC electrodes with improved stability upon extended cycling.

In this work, we present a scalable “top-down” strategy for the synthesis of EC electrode materials by electrochemically expanding micron-scale high temperature-derived layered manganese-rich oxides. Inspired by the electrochemical exfoliation methods developed for large sheet graphite and MoS_2_, this batch process is significantly quicker than traditional chemical exfoliations and produces expanded bulk particles (Ma et al., 2008; Liu et al., 2014; Achee et al., 2018; Ambrosi and Pumera, 2018). We then show that assembly of the micron-sized expanded materials into electrodes leads to high power capacitive energy storage. Comparison with commercial activated carbon shows that the oxides exhibit similar rate capability but higher volumetric capacitance. We hypothesize that the electrochemical expansion enables high power and high volumetric capacitance via two important changes in the oxide: (1) interlayer hydration, which improves interlayer diffusion kinetics while also buffering intercalation-induced structural changes, and (2) particle expansion, which significantly improves electrode integrity and volumetric capacitance.

## Materials and Methods

### Materials Synthesis: Bulk Powders

A coprecipitation and calcination method was used to prepare the micron-scale layered oxide powders with a P2 structure (Boyd et al., 2018). Briefly, a 2:1 ratio of Mn(CH_2_CHOO)_2_·4H_2_O and Cu(CH_2_CHOO)_2_ (Alfa Aesar) were dissolved in deionized water, and added at ~1 drop/s into a stirred aqueous NaOH (Fisher Scientific) solution of pH ~12. After aging for ~1 h, the Mn_0.62_Cu_0.31_(OH)_2_ precipitate was washed until pH neutral, filtered, and dried overnight at 120°C. The hydroxide precursor was ball milled in ethanol for 24 h. After drying the material again, Na^+^ was added in a 0.74:1 molar ratio of Na^+^:MCu(OH)_2_ by mixing the precursor and NaOH in DI water for 1 h at room temperature and then heating it at 100°C on a stir plate until dry. The dried powder was ground with a mortar and pestle to break up any large chunks, and annealed at 500°C for 5 h. After cooling to room temperature, the powder was ground for 15 min before calcination at 850°C for 6 h and subsequent transfer to an inert-atmosphere glovebox (<1 ppm H_2_O and O_2_) while at ~70°C. The calcined powder was ground with a mortar and pestle for 30 min in the glovebox. The composition of the as-synthesized material was Na_0.64_Mn_0.62_Cu_0.31_O_2_, abbreviated NaMCu. A Ni-containing material, Na_0.64_Ni_0.22_Mn_0.66_Cu_0.12_O_2_ (NaNMCu), was synthesized in the same manner.

### Materials Synthesis: Electrochemical Expansion

To electrochemically expand and hydrate the P2 powders, ~0.5 g of the material was placed into an electrolyte-permeable and electronically-connected pouch acting as the positive electrode in a two electrode cell, vs. a Ni foam negative electrode. The pouch was made by folding double-layer dialysis tubing (Thermo Scientific SnakeSkin®, 10,000 MWCO, 22 mm diameter) around a coiled Pt wire electrode (Pine Instruments, 99.99% pure) and sealing it with parafilm (Figure S1). The dialysis tubing held the NaMCu powder near (and optimally in contact with) the Pt wire. After ensuring that the 0.5 M K_2_SO_4_ (Fisher Scientific) electrolyte fully wet the dialysis tubing, +4 V was applied to the cell for 24 h. After expansion, the pouch was rinsed and cut open. The expanded material was washed out of the pouch using DI water, and centrifuged at 4,500 RPM for 3–5 min to remove excess water. The powders were rinsed at least twice more to remove all electrolyte salt, and centrifuged after each rinse. Finally, the expanded powder was air dried at room temperature.

### Methods: Synchrotron X-Ray Diffraction

Synchrotron X-ray diffraction (XRD) was performed at the Stanford Synchrotron Radiation Lightsource (SSRL) on beamline 7–2 with a 14.013 keV beam. The *operando* diffraction patterns were collected with a Pilatus 300 K area detector (DECTRIS Ltd.) in portrait mode, in a Bragg-Brentano (θ-2θ) reflection geometry at a sample-to-detector distance of 750 mm. XRD patterns were taken with the middle of the detector at 11.4 and 23.0°2θ with a 3 s exposure every 20 s while cycling the electrode at 0.5 mV/s. After calibrating the detector geometry (tilts and distance) with LaB_6_, the area diffraction patterns were reduced to one dimensional intensity vs. 2θ patterns with the pyFAI library (Ashiotis et al., 2015). The electrochemical cell consisted of a 1 M Na_2_SO_4_ (Fisher Scientific) electrolyte, a Pt counter electrode, and a miniature leakless Ag/AgCl reference electrode (eDAQ ET072-1) using a cell developed for *in situ* electrochemical X-ray scattering (Cao et al., 2016; Mitchell et al., 2019). The preparation of the working electrode is discussed in more detail in section Methods: Electrochemical Characterization. Briefly, an 8:1:1 slurry of the P2 oxide, acetylene black, and polyvinylidene fluoride was prepared in n-methyl pyrrolidone, cast onto a plasma-cleaned platinized silicon substrate (MTI Corp.), and dried at 120°C overnight.

### Methods: Physical Characterization

XRD was used to determine the structure of the as-synthesized P2 and expanded powders, and the electrodes before and after electrochemical cycling. XRD was performed with standard Bragg-Brentano geometry and Cu-K*a* radiation (PANalytical Empyrean). The powder samples were rotated at 7.5 RPM, while the electrodes remained stationary. Scanning electron microscopy (SEM, FEI Verios 460L) was used to determine the morphology of the P2 powders before and after expansion, as well as after electrochemical cycling. The water content of the expanded samples was measured with thermogravimetric analysis (TGA; SII EXSTAR6000 TG/DTA6200), using aluminum pans to hold ~12 mg of powder during heating in air at 5°C /min from 25 to 300°C.

### Methods: Electrochemical Characterization

#### Electrode Preparation

The electrochemical behavior of the expanded particles was characterized using slurry electrodes cast onto either Ti mesh or foil (Alfa Aesar). The metal substrates were cleaned by sonication in ethanol, after which the mesh electrodes were dried and coated with slurry. The working face of the Ti foil was etched with sandpaper to improve slurry adhesion while the back of the foil electrode was covered with Kapton tape to minimize its current contribution during electrochemical cycling. The electrode was then plasma cleaned (Harrick Plasma PDC-32G) for 3 min also to improve slurry adhesion.

The slurry composition varied depending on the type of current collector (mesh or foil). We found that for the expanded oxide on Ti mesh, a slurry composition of 80 wt% active material, 17.5 wt% acetylene black (Alfa Aesar), and 2.5 wt% polyvinylidene fluoride (PVDF, Arkema Kynar KV 900) gave good adhesion to the mesh and the best rate capability. The acetylene black slurries, used to understand the current contribution from the conductive carbon additive, consisted of 67 wt% acetylene black and 33 wt% PVDF. To compare the electrochemically expanded NaMCu to commercial activated carbon on a volumetric basis, we used foil current collectors. For the activated carbon slurry on Ti foil, 90 wt% YP50 activated carbon (Kuraray Co., Ltd.) was combined with 10 wt% PVDF. The expanded oxide slurry on Ti foil consisted of 80 wt% active material, 15 wt% acetylene black, and 5 wt% PVDF. The dry components of each slurry were ground for 5 min before adding n-methyl pyrrolidone (NMP; Sigma Aldrich) and stirring overnight on a magnetic stir plate at 990 RPM. After painting the slurry onto the current collector, the electrodes dried for several hours at room temperature in the fume hood. At this point, the foil electrodes were placed between a folded weigh paper and calendared to the approximate thickness of the electrode-weigh paper assembly with a rolling mill (Durston) before transfer to an oven at 120°C. We transferred the mesh electrodes directly from the fume hood to the oven at 120°C, where they dried for several hours before calendaring to 125 μm between the sheets of a folded weigh paper. The final thickness of the mesh electrodes was ~90 μm.

#### Electrochemistry

All electrodes were tested with a three-electrode set up in glass 50 mL round bottom flasks with an aqueous 0.5 M K_2_SO_4_ (Fisher Scientific) electrolyte, using an Ag/AgCl in 3.5 M KCl reference electrode (Pine Instruments) and a platinum wire counter electrode (99.997%, Alfa Aesar). All tests used Ni-plated stainless steel alligator clips to hold the working and counter electrodes in the electrolyte. The cyclic voltammetry was performed with a Biologic MPG2 potentiostat.

## Results and Discussion

### Mechanism of Electrochemical Expansion

**Figure 1:**
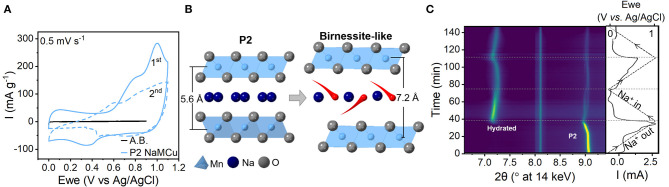
Mechanism of water intercalation into P2-type NaMCu upon electrochemical cycling in 1 M Na_2_SO_4_. **(A)** Cyclic voltammetry at 0.5 mV/s. A.B. indicates “acetylene black,” the electrochemical response of the conductive carbon in the electrode. **(B)** Cartoon showing the insertion of water molecules (red) from the electrolyte into the material interlayer to compensate for the electrochemical de-intercalation of Na^+^ (dark blue). **(C)** *Operando* synchrotron XRD during the first two CV cycles at 0.5 mV/s. The right-most panel shows the applied potential (dashed line) and the current response (solid line). During the first anodic cycle, the interlayer peak of the hydrated birnessite-like phase (~7°2θ) emerges around 0.9 V. Both the P2 and the hydrated peak show reversible, continuous changes in the interlayer spacing as a function of potential attributed to Na^+^ de/intercalation. The peak at 8.1°2θ is due to the electrochemical cell.

To determine the mechanism of electrochemical expansion and interlayer hydration of P2-type layered sodium manganese-rich oxides in aqueous electrolytes, we performed *operando* XRD during cyclic voltammetry of NaMCu and NaNMCu. The crystal structure of a P2-type layered oxide (Figure S2A) consists of layers of edge-sharing *M*O_6_ octahedra, and ~67% of the interlayer prismatic sites are filled with Na^+^ (Delmas et al., 1980). Figure 1A shows the cyclic voltammogram (CV) of P2 NaMCu at 0.5 mV/s in 1 M Na_2_SO_4_. During the first anodic cycle, the CV of NaMCu shows current peaks around 0.2 and 0.9 V vs. Ag/AgCl. On the subsequent cathodic scan, the overall current decreases and is relatively constant as a function of potential. These electrochemical changes suggest a change in the structure and energy storage mechanism in the material, which we attribute to expansion (*vide infra*) and interlayer water insertion as shown in Figure 1B and described in our previous work (Boyd et al., 2018). Well-defined current peaks correlate to a well-defined Gibbs free energy of reaction, which in an intercalation-type material indicates little dispersion in the intercalation site energy. On the other hand, a constant current as a function of potential, such as observed after the first anodic sweep, is indicative of a capacitive mechanism exhibited by many layered manganese oxides in neutral pH electrolytes (Brousse et al., 2006; Ghodbane et al., 2012; Leong and Yang, 2019). The capacitive charge storage mechanism of manganese oxides under these conditions is a subject of debate, but has been attributed to the correlated intercalation of cations and water molecules (Bélanger et al., 2008; Arias et al., 2014; Costentin et al., 2017).

The structural changes of NaMCu during the first two CV cycles were measured with *operando* synchrotron XRD (Figure 1C). Two changes are apparent: the appearance of a new peak during the first anodic cycle (discussed below) and shift of the NaMCu diffraction peak. The interlayer spacing of the P2 phase, indicated by the (002) peak at ~9°2θ, increases from 5.59 Å (9.07°2θ) at 0 V to 5.65 Å (8.97°2θ) at 1.1 V. Upon reduction to 0 V, the spacing returns to nearly its original value, 5.61 Å (9.04°2θ). This shows that the interlayer spacing reversibly increases during oxidation and decreases during reduction. The continuous change of the NaMCu interlayer spacing as a function of potential indicates a solid-solution intercalation mechanism, similar to its structural behavior in non-aqueous Na^+^ electrolytes (Kang et al., 2015; Li et al., 2015; Wang L. et al., 2017). The faint splitting of the P2 (001) peak near the end of the cathodic cycle may indicate that the remaining P2 material forms two phases with slightly different Na^+^ content.

In addition to this expected behavior, the appearance of a new peak at 7.19°2θ at ~0.9 V during the first anodic cycle indicates the formation of a new phase. According to prior results, this peak corresponds to the (001) plane of a birnessite-like hydrated phase with an interlayer spacing of 7.05 Å (Lu and Dahn, 2001; Abou-El-Sherbini et al., 2002; Buchholz et al., 2014; Boyd et al., 2018; Yao et al., 2019). The full XRD patterns of the material before and after the phase transformation are shown in Figure S2B. The large interlayer spacing of the new phase indicates the insertion of a single layer of water molecules from the aqueous electrolyte into the interlayer of NaMCu (Nam et al., 2015; Li et al., 2016). Moreover, reversible peak shifts indicate that this new hydrated phase is electrochemically active: upon reduction from 1.1 to 0 V, the interlayer spacing decreases to 6.97 Å (7.27°2θ). During the second cycle it increases to 7.07 Å (7.17°2θ) at 1.1V and shifts back to 6.97 Å (7.27°2θ) at 0 V. This change of ~ 0.1 Å is about half of that typically observed in birnessite in aqueous electrolytes (Ghodbane et al., 2012; Xiong et al., 2017). The reversible peak shifts of the hydrated phase indicate that cation (de)intercalation into the hydrated interlayer at least partially contributes to the capacitive energy storage mechanism. These changes would not appear if the capacitive behavior was simply due to the formation of the double layer at the outer surface of the particles. The intensity increase of the hydrated phase peak and corresponding decrease in the P2 (002) peak during the first anodic sweep show the partial phase transformation of the electrode material. However, all peak intensities decrease after ~0.5 V during the second cathodic sweep, as the change in particle volume during electrochemical expansion causes the electrode to delaminate from the current collector.

Figure S3 shows similar behavior in a P2 oxide with a different composition, NaNMCu, where the inclusion of nickel led to Na^+^ deintercalation and the subsequent formation of the birnessite-like phase at a lower potential (Kang et al., 2015; Wang L. et al., 2017; Chen et al., 2018). Overall, the *operando* results show that when these P2 oxides are cycled in an aqueous electrolyte, water incorporation occurs during Na^+^ deintercalation, likely to offset the increasing electrostatic repulsion between the transition metal and oxygen layers. Within an individual particle, the formation of this birnessite-like phase may lead to the capacitive CV observed after the first cycle. However, the large expansion of the P2 particles (25% *c*-axis increase) in the slurry electrode leads to loss of electronic connection to the current collector, or even delamination, when cast onto the electrode before expansion. If the electrochemical expansion is better controlled, the formation of micron-scale particles with hydrated interlayers with a capacitive electrochemical response indicates that these particles could be viable for high volumetric capacitance EC materials.

### Batch Expansion Process

**Figure 2:**
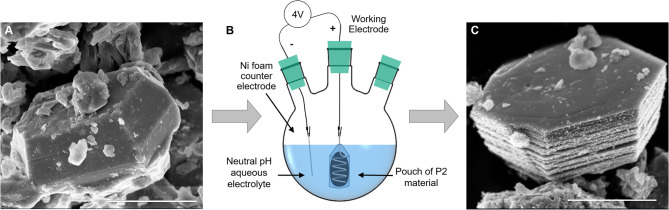
Effect of batch expansion on particle morphology: **(A)** a pristine P2 NaMCu particle has a compact structure, which expands after electrochemical water insertion. **(B)** A schematic of the electrochemical expansion setup, where the P2 material is contained in a dialysis tubing pouch around a coil of Pt wire, and the counter electrode is Ni foam. **(C)** A typical fully expanded NaMCu particle, which retains its *ab*-plane morphology but expands vertically due to water insertion. Scale bar 2 μm.

**Figure 3:**
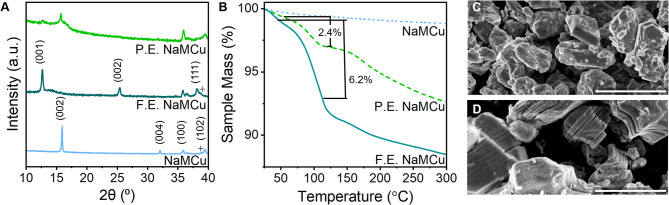
Structural characterization of expanded NaMCu materials. **(A)** Powder XRD shows the complete transformation of the F.E. material into the hydrated phase, while the P.E. material contains both the hydrated phase and residual P2 phase. **(B)** TGA shows that both F.E. and P.E. NaMCu materials lose a significant amount of water with increasing temperature. SEM of the **(C)** pristine NaMCu powder and **(D)** F.E. NaMCu powder shows that the expanded particles retain their initial hexagonal morphology and particle size but are now expanded along the layers. Scale bar 3 μm.

To circumvent the electrical contact and slurry delamination problems resulting from *in situ* P2 particle expansion on electrodes, we developed a batch expansion process for the P2 oxide powder, as described in the Experimental section. Figure 2 shows the expansion cell, along with scanning electron microscopy (SEM) images of a P2 particle (Figure 2A) and an expanded particle (Figure 2C). The resulting expanded and hydrated particles can be assembled into electrodes, thus bypassing the issue of electrode delamination after direct electrochemical expansion of the P2 particles. The batch process uses a neutral pH electrolyte, a voltage source, permeable membrane, and a Ni foam counter electrode, rendering it environmentally friendly and scalable. Figure S4 shows representative chronoamperometry for the process. We observed that a smoother current response is a good indicator of expansion quality—noisier curves resulted in less expansion of the P2 oxide, as indicated by XRD (Figure 3A). This is likely due to the variability of the electrical contact between the powders and the Pt coil in the pouch cell, which could be improved with more advanced pouch designs. This synthesis method offers a new, scalable method to produce materials for EC electrodes that can provide high volumetric capacitance.

### Structural Characterization of the Expanded Materials

To determine the extent to which the batch expansion process affects material structure and electrochemical performance, we compared three structures: as-synthesized NaMCu, fully expanded (F.E.) NaMCu, and partially expanded (P.E.) NaMCu. Figure 3 shows the XRD, TGA, and SEM of these materials. The pristine NaMCu has the same structure as reported previously (Boyd et al., 2018): a primary P2-type phase (^*^) and a small amount of CuO (+). The first peak at 15.92°2θ (5.57 Å) corresponds to the (002) peak of the P2 phase. The F.E. NaMCu shows a completely expanded and hydrated structure: the P2-phase is no longer present and the interlayer peak at 12.99°2θ (6.99 Å) indicates a 25% expansion, the same as the *operando* XRD experiments. As discussed above, the large increase in interlayer spacing is due to the transformation to a new hydrated phase. Both the P2 and F.E. NaMCu materials exhibit a peak at 35.8°2θ (2.5 Å). This peak corresponds to the (100) plane of the P2 structure, and its presence in both the pristine and F.E. materials indicates that the lattice spacing along the transition metal oxide sheets is unaffected by electrochemical expansion. The larger *c*-axis in the expanded materials leads to slightly decreased angles of the (101) and (102) peaks at 36.5 and 38.1°2θ. The two-phase P.E. structure indicates that the expansion proceeds via a direct phase transformation and agrees with the *operando* XRD, which shows the emergence of the hydrated phase at a distinct potential as opposed to a gradual structural transition over a broad potential range.

TGA of each material indicates decreasing mass loss between room temperature (~25°C) and 300°C in the order F.E. NaMCu > P.E. NaMCu > NaMCu. In this temperature range, mass losses are most likely due to the removal of water molecules from the oxide surface and interlayer. The composition of the F.E. material for TGA was Mn_0.62_Cu_0.31_O_2_·*x*H_2_O, assuming the removal of all Na^+^ during electrochemical expansion. This means that the 6.2 wt% lost from F.E. NaMCu between 50 and 120°C corresponds to 0.33 mol H_2_O per MCu (*x* = 0.33). Many birnessites also have a oxide:water content ratio near 1:0.33, supporting the XRD data that electrochemical expansion leads to insertion of a single layer of water molecules into the interlayer of the P2 oxides. The P.E. NaMCu lost 2.4 wt%, which suggests only ~ 38% of the powder expanded based on the mass lost from F.E. NaMCu. Lastly, NaMCu exhibits only a minor 1.2 wt% loss, likely due to the desorption of surface water.

The SEM images in Figures 3C,D show that the crystallographic change affects the morphology as well: the *ab* plane particle size and shape are retained after expansion, while the *c*-axis dimensions increase as the layers experience varying degrees of partial exfoliation. Figure 2 shows higher magnification SEM images of pristine and expanded NaMCu particles. Before expansion, the material consists of dense, roughly hexagonal, micron-scale primary particles. Based on the crystal structure, the interlayer spacing is parallel to the surface of hexagon-like primary particles. After electrochemical oxidation, there is clear partial exfoliation of the particles along the *c* axis that gives rise to large pores hundreds of nanometers in dimension. We hypothesize that the large pores and interlayer hydration formed by the expansion process enable high power energy storage by increasing access of electrolyte ions to the interlayer and buffering the structural changes during ion (de)intercalation.

### Electrochemical Behavior of Expanded Materials

**Figure 4:**
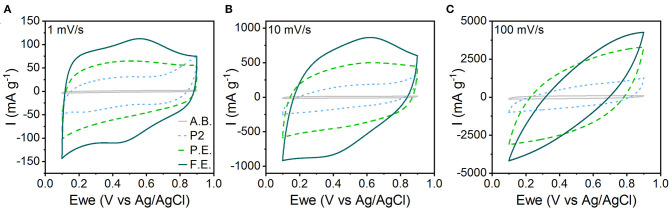
CVs of the pristine P2, P.E., and F.E. NaMCu materials in 0.5 M K_2_SO_4_ electrolyte at **(A)** 1 mV/s, **(B)** 10 mV/s, and **(C)** 100 mV/s. The specific current from just the acetylene black (A.B.) conductive additive is shown for reference.

Electrochemical characterization shows that the capacitive character of the material increases with amount of interlayer hydration and expansion. We characterized the electrochemical behavior of pristine, P.E., and F.E. NaMCu in a 0.5 M K_2_SO_4_ electrolyte. This electrolyte was selected over Na_2_SO_4_ because K^+^ has a smaller hydrated ion radius compared to Na^+^, which is hypothesized to allow higher rate capability and cyclability in aqueous electrolytes (Shao et al., 2013; Xiong et al., 2017; Dupont et al., 2018; Phadke et al., 2020). Figure 4 shows the CVs of the pristine, P.E., and F.E. NaMCu in 0.5 M K_2_SO_4_ at 1, 10, and 100 mV/s. The response of the acetylene black conductive additive is also shown for reference. It contributes only minimal double-layer current. The F.E. NaMCu exhibits a capacitive CV at 1 and 10 mV/s, with a semi-rectangular shape and broad peaks similar to those often observed in birnessite (Xiong et al., 2017; Yao et al., 2019). The P.E. NaMCu also has a semi-rectangular CV, albeit with a lower specific current and lacking the broad peaks present in the F.E. material. In contrast, the pristine P2 NaMCu particles expand upon intercalating water during the first anodic cycle, and the electrode capacity drops significantly as the 25% increase along the *c*-axis causes particles to delaminate. P2 NaMCu electrodes do not show clear redox peaks in K_2_SO_4_ (Figure S5), possibly because the interlayer Na^+^ partially exchanges with K^+^ and H_2_O immediately upon immersion into the electrolyte. The semi-rectangular CV, with the lowest specific current of the three electrodes, is likely from the response of the remaining, partially-hydrated material that is still in electrical contact with the current collector. By 100 mV/s, all three materials exhibit significant polarization arising from increased Ohmic resistance from the large measured currents. Overall, these results show that the degree of expansion directly determines the material's electrochemical behavior, and that electrochemical expansion before electrode assembly leads to stable, capacitive energy storage.

**Figure 5:**
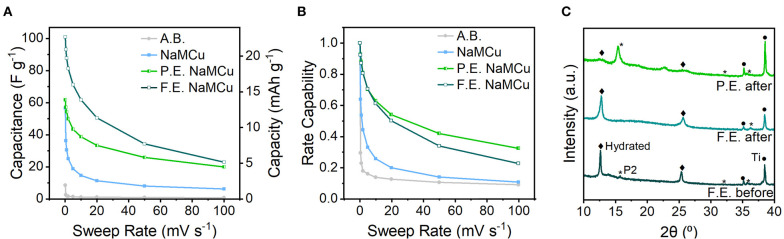
Electrochemical energy storage rate capability and post-electrochemical cycling material structure. **(A)** Cathodic capacitance and **(B)** rate capability (cathodic capacitance normalized to the capacitance at 0.1 mV/s), from 0.1 to 100 mV/s. The low capacity and rate capability of the acetylene black (A.B.) conductive additive shows that the capacitive electrochemical response is due to the oxide active materials. **(C)** *Ex situ* XRD of electrodes after electrochemical cycling shows that the F.E. and P.E. retain their initial structures.

Figure 5 shows the cathodic capacitance and rate capability of the three materials. The highest capacitance of 102 F/g (23 mAh/g) is obtained with the F.E. materials, indicating that the interlayer expansion enabled access of electrolyte ions to the interlayer. This value is comparable to capacitances of activated carbon materials, which is surprising given the much smaller surface area of the oxide materials. When cycled from 0.1 to 50 mV/s, the F.E. NaMCu retains 34% of its initial capacitance. The maximum capacitance of the P.E. NaMCu is lower, 62.4 F/g (14 mAh/g), but its rate capability is slightly better than that of F.E. NaMCu. The pristine P2 NaMCu undergoes electrochemical expansion and hydration directly on the electrode, and its second-cycle capacitance of 57 F/g (13 mAh/g) drops off significantly as the material delaminates from the electrode. *Ex situ* XRD (Figure 5C) indicates that F.E. and P.E. NaMCu retain their initial structure after electrochemical cycling. There is no *ex situ* XRD of the P2 electrodes after electrochemistry because the slurries delaminated from the current collectors during cycling. Together, these results show that the F.E. and P.E. NaMCu exhibit relatively high capacitance and rate capability for aqueous energy storage due to the hydrated interlayer and particle expansion. The batch electrochemical expansion of P2 NaMCu and subsequent electrode assembly leads to electrodes with high rate capability and is promising for high-power energy storage.

**Figure 6:**
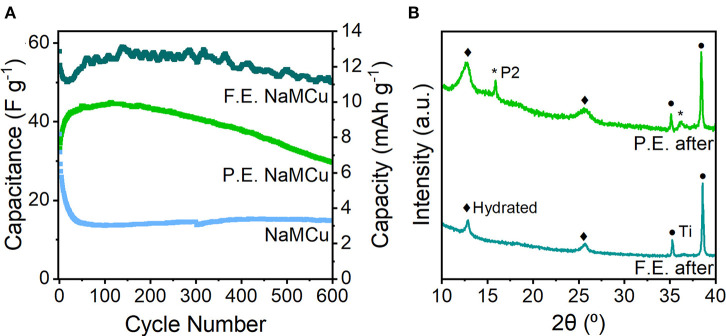
**(A)** Cycling stability of F.E., P.E., and P2 NaMCu over 600 cycles at 10 mV/s. **(B)** *Ex situ* XRD after 600 cycles shows that the F.E. NaMCu retains the hydrated phase, while some of the remaining P2 material in the P.E. NaMCu electrode expanded after prolonged cycling.

Evaluation of the cyclability of the three oxide materials shows that the F.E. NaMCu electrode retained its structure and the most capacity after 600 cycles at 10 mV/s (Figure 6), while the P.E. NaMCu converted to the F.E. structure and the P2 NaMCu delaminated from the electrode. The initial capacitances were 58 F/g (13 mAh/g) for the F.E. electrode, 34 F/g (8 mAh/g) for the P.E. electrode, and 37 F/g (8 mAh/g) for the P2 electrode. After 600 cycles at 10 mV/s, the F.E. electrode retained 86.3% capacity, the P.E. retained 85.7%, and the P2 retained 40.4%. Figure 6 shows that the F.E. electrode experienced an initial capacity drop, but after 60 cycles the capacity increased close to its initial value. The P.E. electrode experienced a steady capacity increase over the first 50 cycles, and then a steady decline during the subsequent 550. The P2 NaMCu lost most of its capacity within the first 50 cycles, as water insertion into the P2 NaMCu partially expanded the oxide particles, causing them to fall off the electrode due to the change in volume. The materials with an expanded and hydrated interlayer exhibit a significant improvement over the as-synthesized P2 materials, likely due to the improved access of the electrolyte to the material. However, further investigation is required to understand the reaction mechanisms and develop their electrode architecture so they can withstand the ~1,000,000 cycles required of ECs (Conway, 1999; Nybeck et al., 2019).

XRD of the F.E. and P.E. NaMCu after the cyclability testing (Figure 6B) shows that the F.E. phase is stable under these electrochemical conditions, as the only visible change is a slight broadening of the peaks after cycling. This suggests that the particle expansion and interlayer hydration limit further structural changes in the material, leading to better stability. *Ex situ* XRD of the P.E. material confirms that an increasing fraction of the F.E. material develops after extended cycling. It appears that the initial fraction of expanded particles was enough to maintain electronic connectivity with the particles expanded *in situ*. Slurry delamination during cycling prevented *ex situ* post-cycling XRD of the pristine NaMCu, but our previous work showed that after 50 cycles, NaMCu partially transformed to the hydrated phase (Boyd et al., 2018).

**Figure 7:**
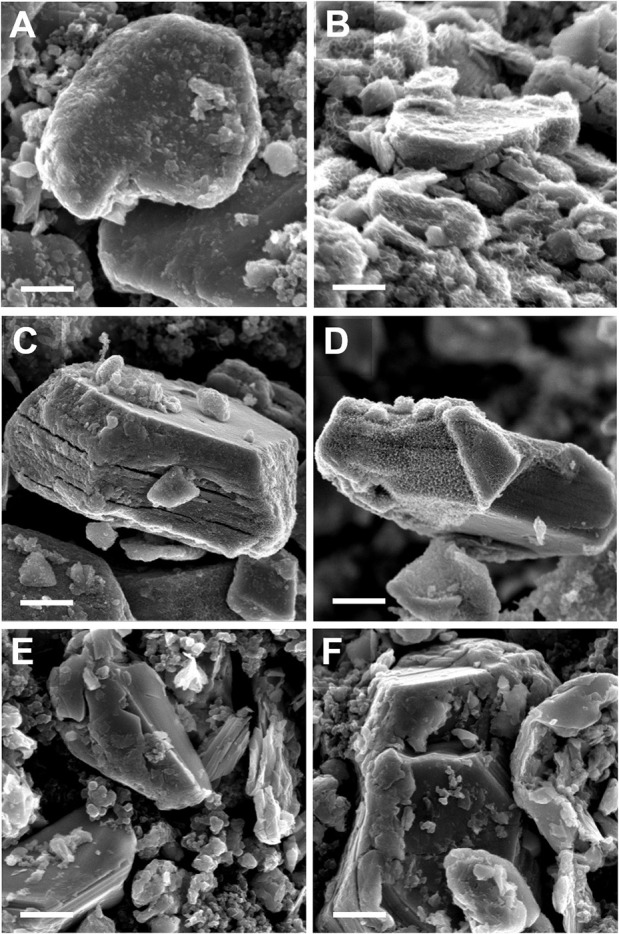
SEM images of **(A,B)** F.E., **(C,D)** P.E., and **(E,F)** P2 NaMCu electrodes after 600 cycles in 0.5 M K_2_SO_4_. While most F.E. particles show expansion or a surface coating, few P.E. particles show surface coating, and few P2 particles even show expansion. Scale bar 500 nm.

Post-electrochemical SEM (Figure 7) shows that while most particles in the F.E. and P.E. electrodes retained their original morphology, some exhibit a nanostructured surface coating. After 600 cycles, the morphology of the F.E. NaMCu primary particles appears similar as before cycling. However, the surface of these primary particles now has a nano–structured, semi-porous coating. One possible mechanism of this surface coating could be restructuring due to the dissolution of Mn^2+^ during reduction, and subsequent re-deposition during oxidation (Liang et al., 2019). The variability of this surface coating from particle to particle in F.E. NaMCu may highlight how the electrode architecture affects the relevant electrochemical reaction. A well-connected particle may experience more overall charge transfer, leading to surface restructuring. However, there was little surface coating in the P.E. NaMCu, suggesting that more of the current may have gone into expanding the remaining P2 particles. Figure 7D shows one of the few P.E. NaMCu particles with partial surface coating. Finally, Figures 7E,F show the pristine P2 NaMCu after 600 cycles. While the P2 particles expanded somewhat, their morphology is much smoother than F.E. or P.E. electrode particles. The presence of only F.E. and Ti substrate peaks in the XRD results confirm that this surface film is either the same hydrated birnessite-like phase or it is just a minor weight fraction of the total electrode mass. Overall, the electrochemistry, XRD, and SEM results highlight that electrodes assembled from pre-expanded F.E. or P.E. NaMCu possess high rate capability and structure stability that make them suitable as EC materials.

### Comparison of Expanded NaMCu to Commercial Activated Carbon

**Figure 8:**
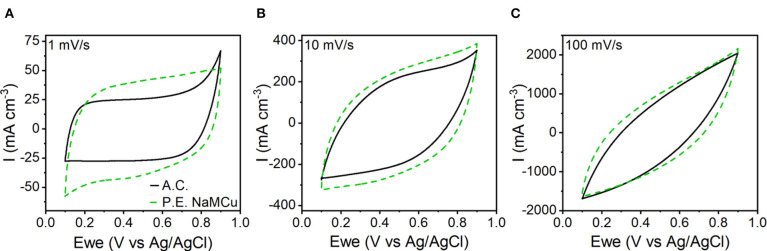
CV comparison of P.E. NaMCu and activated carbon (A.C.) on the basis of their specific current at **(A)** 1 mV/s, **(B)** 10 mV/s, and **(C)** 100 mV/s. At sweep rates up to ~20 mV/s, the P.E. NaMCu out-performs the activated carbon, although both electrodes appear relatively resistive at 100 mV/s.

**Figure 9:**
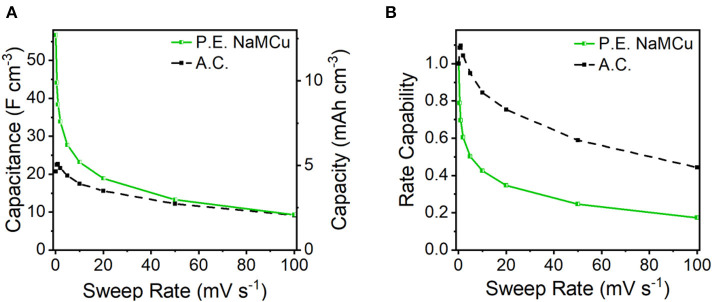
**(A)** Volumetric capacity/capacitance and **(B)** rate capability of P.E. NaMCu and activated carbon (A.C.) electrodes.

To test our hypothesis that the hydrated and expanded NaMCu would allow an increase in the volumetric capacitance of EC electrodes by increasing access of electrolyte ions to the interlayer and buffering structural changes associated with ion (de)intercalation, we compared the P.E. NaMCu with a commercially available activated carbon used for EC electrodes. P.E. NaMCu was selected due to its better rate capability than F.E. NaMCu. To enable comparison of the volumetric capacity/capacitance, the materials were cast onto foil (vs. mesh) current collectors. Figure 8 compares the CVs for both materials at 1, 10, and 100 mV/s in 0.5 M K_2_SO_4_ while Figure 9 shows the specific capacity/capacitance and rate capability. Both P.E. NaMCu and activated carbon exhibit nearly rectangular, ideally capacitive CVs. At sweep rates up to 20 mV/s (corresponding to a charge/discharge time of 40 s), P.E. NaMCu shows a higher specific current than activated carbon, indicating that the expanded oxide particles give better volumetric performance than the high surface area activated carbon. At 0.1 mV/s, the volumetric capacitances of P.E. NaMCu and activated carbon are 57.2 F/cm^3^ (12.7 mAh/cm^3^) and 20.9 F/cm^3^ (4.64 mAh/cm^3^), respectively. However, the response of the two materials becomes similar at faster sweep rates; by 100 mV/s the CVs essentially overlap and their diagonality indicates both are more Ohmically resistive. The similarity of the material response at faster sweep rates may be due to similar issues of maintaining adequate ionic and electronic conductivity in the electrodes. These results show that an oxide material with expanded and hydrated micron-size particles can increase EC energy density over traditionally used high surface area activated carbon at timescales of up to 40 s. While further studies are required to determine the exact mechanism, we propose that the expanded and hydrated interlayer facilitates access of electrolyte ions for charge storage, enabling better material utilization at high rates.

## Conclusions

This work describes a promising new material for EC electrodes by detailing how electrochemically expanding micron-sized layered oxides and hydrating the interlayer leads to EC electrodes with high volumetric capacitance and cyclability in aqueous electrolytes. We propose a scalable “top-down” synthesis to electrochemically expand particles, changing their electrochemical characteristics from battery-like for the initial Na^+^ deintercalation to capacitive upon subsequent cycling. Specifically, after interlayer hydration and subsequent particle expansion, micron-sized P2 oxide particles exhibit capacitive behavior that is at least in part pseudocapacitive, as evidenced by reversible interlayer spacing changes during *operando* XRD. The obvious reduction in redox peaks in CVs after expansion suggests that the material deformation is further buffered by the interlayer water. In addition, the surface area of the bulk expanded particles is only slightly greater than that of the pristine materials. Therefore, we hypothesize that the interlayer expansion and hydration effectively increase the surface area by extending the electrolyte into the bulk of the particles in two ways. First, hydrating the material interlayer improves cation transfer from the bulk electrolyte to the particles, and second, the large (~100 nm) pores between expanded sections of the material allow easier access of the bulk electrolyte to the particles. The hydrated interlayers may also cushion the structural effects of ion intercalation. All of these increase the rate at which electrolyte cations can approach transition metal oxide storage sites. These results show that hydrating the material interlayer allowed the micron-scale particles to maintain their structure during extended cycling, making them compatible for capacitive energy storage in aqueous electrolytes. By expanding the interlayer of P2 NaMCu oxide, we show that particles with a large solid-state cation diffusion distance can be competitive with the state-of-the-art activated carbon for EC electrodes in aqueous electrolytes. While this synthesis is conducted on P2 oxides, it can be applied to other layered materials that possess an electrochemically labile ion. Overall, this shows that electrochemically-driven expansion of oxide materials can tune both the interlayer chemistry and particle size, leading to a new scalable synthesis strategy for EC materials with high volumetric capacitance.

## Data Availability Statement

The raw data supporting the conclusions of this article will be made available by the authors, without undue reservation.

## Author Contributions

SB and VA contributed to the study conception and design. All authors designed the synchrotron experiments. NG extracted and analyzed the synchrotron data. SB performed all other experiments and performed the data analysis. All authors contributed to writing and revising the manuscript, and read and approved the submitted version.

## Conflict of Interest

The authors declare that the research was conducted in the absence of any commercial or financial relationships that could be construed as a potential conflict of interest.
