# Supplementary material for: High Power Energy Storage via Electrochemically Expanded and Hydrated Manganese-Rich Oxides
Source: Front Chem. 2020 Aug 18;8:715. doi: 10.3389/fchem.2020.00715 (PMC7461800; doi:10.3389/fchem.2020.00715)
Supplement: Supplementary file 1 [file Table_1.DOCX]

Supplementary Material


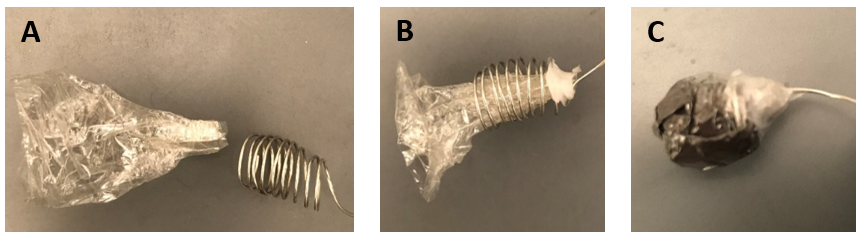


**Supplementary Figure 1.** Fabrication of the expansion pouch. **(A)** Dialysis tubing folded over on itself to form a double-layer wall, with one end folded into a point to thread through the Pt wire coil. **(B)** The threaded dialysis tubing with parafilm securing the folded end to the Pt wire. **(C)** After folding the tubing over the Pt wire and filling the pouch with P2 NaMCu powder, parafilm secures the outer wall of the pouch as well.


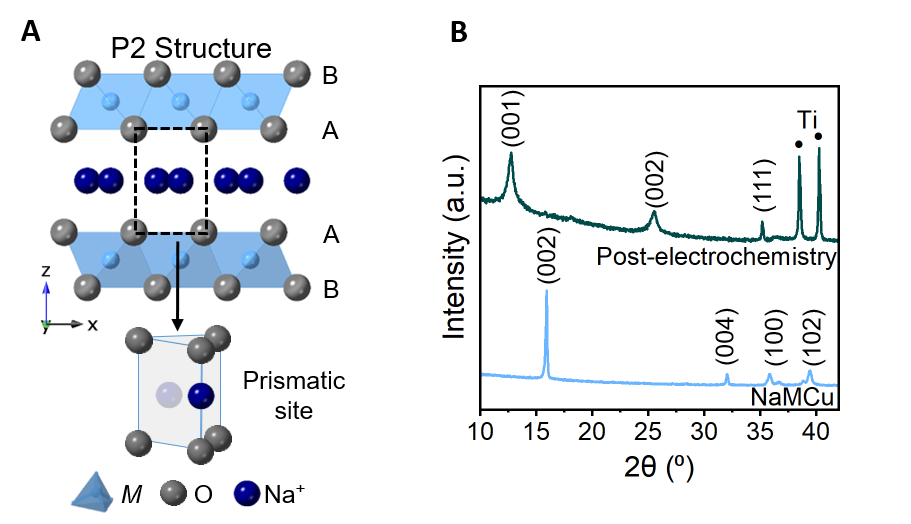


**Supplementary Figure 2.** The P2 structure of the pristine material. **(A)** Model showing the edge-sharing *M*O_6_ octahedra forming layers with … ABBA … oxygen stacking, where *M* represents Mn and other transition metals. The Na^+^ are located in two types of trigonal prismatic sites between the oxide layers. **(B)** XRD pattern of the pristine NaMCu powder (“NaMCu”), where the (002) peak indicates the interlayer spacing. Post-electrochemistry, the NaMCu transforms to a hydrated structure. The substrate peaks of the Ti current collector are indicated by •.

**
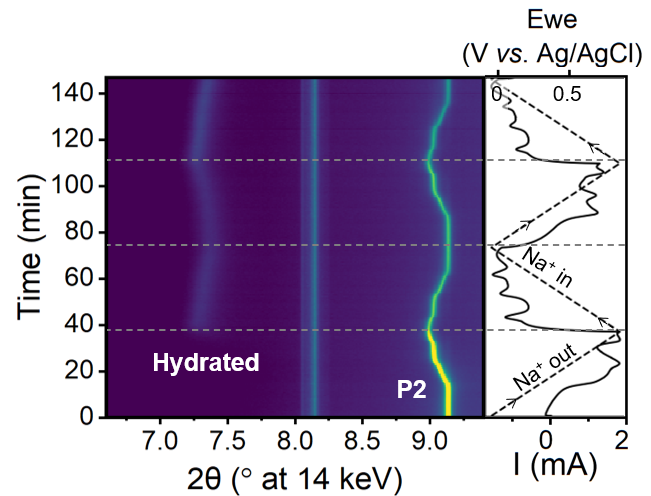
**

**Supplementary Figure 3.** *Operando* synchrotron XRD of NaNMCu, showing the P2 (002) peak ~ 9.11 °2θ, which decreases with material oxidation and Na^+^ extraction up to 0.8 V, and returns to its original position upon reduction to 0 V. This material also experiences the emergence of the hydrated phase at ~ 7.23 °2θ, which continuously contracts (expands) during reduction (oxidation) due to interlayer cation intercalation (deintercalation). Both phases remain electrochemically active during the second cycle, showing reversible changes in interlayer spacing.

**
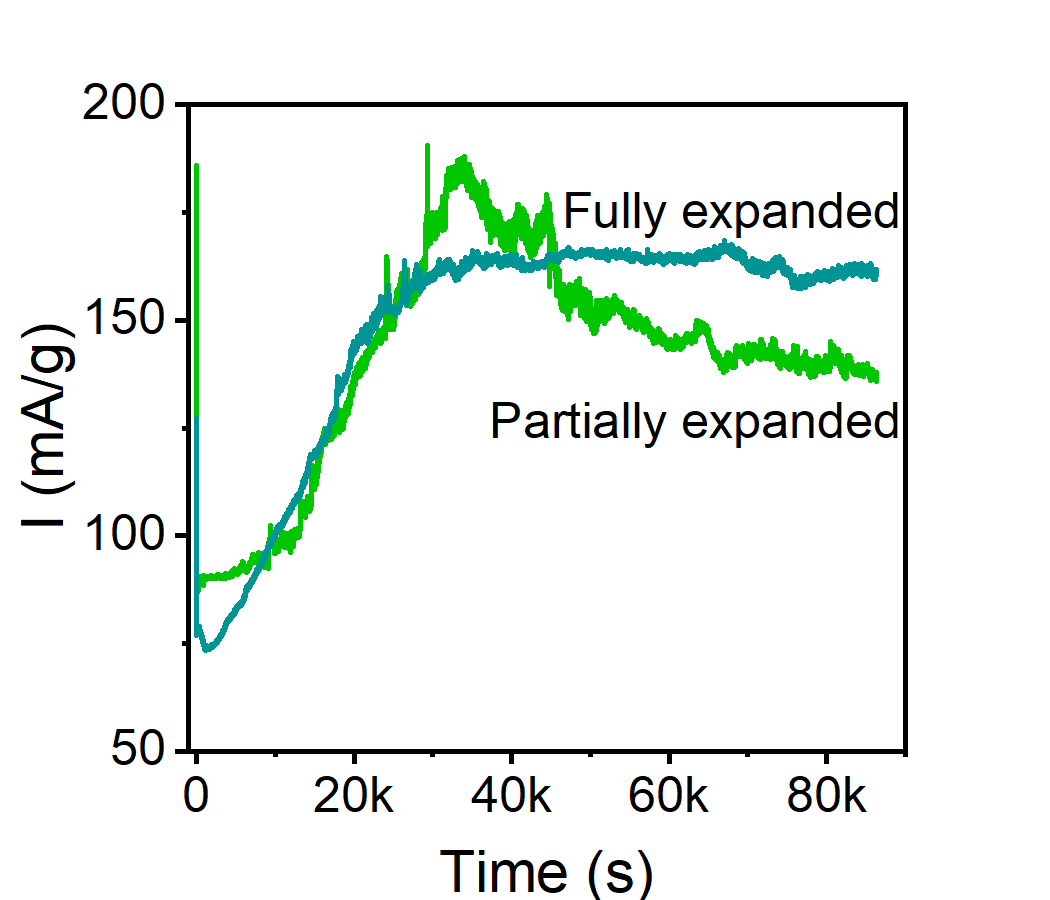
**

**Supplementary Figure 4.** Chronoamperometric expansion of the fully (dark green; F.E.) and partially (light green; P.E.) expanded materials. The “noise” of each curve could be related to the electronic contact of particles within the pouch that determines the extent of electrochemical expansion.

**
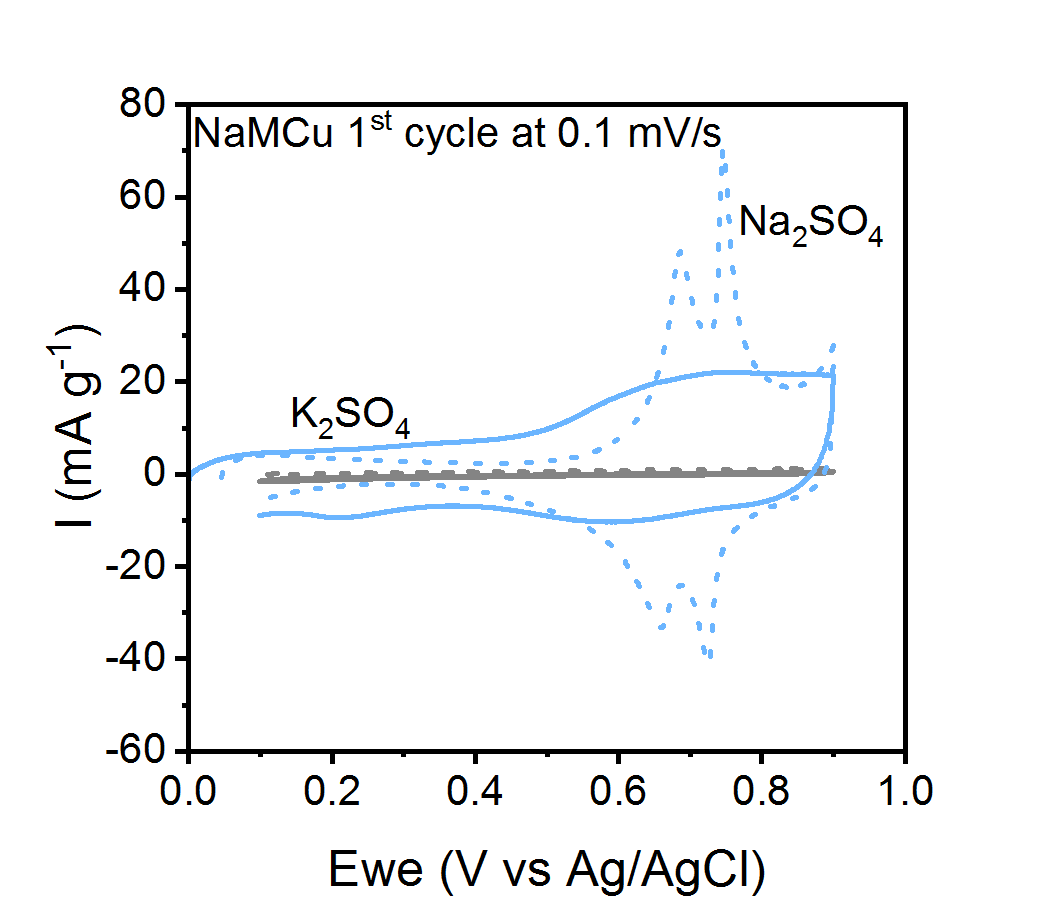
**

**Supplementary Figure 5.** Cyclic voltammograms of the first cycle of pristine NaMCu mesh electrodes in Na_2_SO_4_ (dotted line) and K_2_SO_4_ (solid line) aqueous electrolytes at 0.1 mV/s. Here, the two primary redox peaks of NaMCu in Na_2_SO_4_ occur before 0.8 V vs. Ag/AgCl. Since the hydrated phase in Figure 1 appears around 0.9 V, this suggests that completion of the redox reactions associated with the two anodic redox peaks leads to the intercalation of water and subsequent material expansion.
